# Mortality in East African shorthorn zebu cattle under one year: predictors of infectious-disease mortality

**DOI:** 10.1186/1746-6148-9-175

**Published:** 2013-09-08

**Authors:** Samuel M Thumbi, Mark BMdec Bronsvoort, Henry Kiara, PG Toye, Jane Poole, Mary Ndila, Ilana Conradie, Amy Jennings, Ian G Handel, JAW Coetzer, Johan Steyl, Olivier Hanotte, Mark EJ Woolhouse

**Affiliations:** 1 Centre for Infectious Diseases, University of Edinburgh, Ashworth Laboratories, Kings Buildings, West Mains Road, Edinburgh EH9 3JT, UK; 2 The Roslin Institute, Easter Bush, University of Edinburgh, Roslin, Midlothian, EH25 9RG, Edinburgh, UK; 3 International Livestock Research Institute, P.O. Box 30709, Nairobi 00100, Kenya; 4 University of Nottingham, University Park, Nottingham NG7 2RD, UK; 5 Department of Veterinary Tropical Diseases, Faculty of Veterinary Science, University of Pretoria, Private bag X04, Onderstepoort, South Africa; 6 Department of Paraclinical Sciences, Faculty of Veterinary Science, University of Pretoria, Private bag X04, Onderstepoort, South Africa; 7 Paul G. Allen School for Global Animal Health, Washington State University, Pullman, WA 99164-7090, USA; 8 Kenya Medical Research Institute/CDC Public Health and Research Collaboration, P.O BOX, 1578, Kisumu 40100, Kenya

**Keywords:** Mortality, Infectious-disease, Cattle

## Abstract

**Background:**

Infectious livestock diseases remain a major threat to attaining food security and are a source of economic and livelihood losses for people dependent on livestock for their livelihood. Knowledge of the vital infectious diseases that account for the majority of deaths is crucial in determining disease control strategies and in the allocation of limited funds available for disease control. Here we have estimated the mortality rates in zebu cattle raised in a smallholder mixed farming system during their first year of life, identified the periods of increased risk of death and the risk factors for calf mortality, and through analysis of post-mortem data, determined the aetiologies of calf mortality in this population. A longitudinal cohort study of 548 zebu cattle was conducted between 2007 and 2010. Each calf was followed during its first year of life or until lost from the study. Calves were randomly selected from 20 sub-locations and recruited within a week of birth from different farms over a 45 km radius area centered on Busia in the Western part of Kenya. The data comprised of 481.1 calf years of observation. Clinical examinations, sample collection and analysis were carried out at 5 week intervals, from birth until one year old. Cox proportional hazard models with frailty terms were used for the statistical analysis of risk factors. A standardized post-mortem examination was conducted on all animals that died during the study and appropriate samples collected.

**Results:**

The all-cause mortality rate was estimated at 16.1 (13.0-19.2; 95% CI) per 100 calf years at risk. The Cox models identified high infection intensity with *Theileria* spp., the most lethal of which causes East Coast Fever disease, infection with *Trypanosome* spp., and helminth infections as measured by *Strongyle* spp. eggs per gram of faeces as the three important infections statistically associated with infectious disease mortality in these calves. Analysis of post-mortem data identified East Coast Fever as the main cause of death accounting for 40% of all deaths, haemonchosis 12% and heartwater disease 7%.

**Conclusion:**

The findings demonstrate the impact of endemic parasitic diseases in indigenous animals expected to be well adapted against disease pressures. Additionally, agreement between results of Cox models using data from simple diagnostic procedures and results from post-mortem analysis underline the potential use such diagnostic data to reduce calf mortality. The control strategies for the identified infectious diseases have been discussed.

## Background

Calf mortality is a significant source of economic loss in the livestock industry. In smallholder mixed crop-livestock production systems, the survival of female calves is required for herd expansion and breed improvement, while that of male calves is used as a source of income from sales or as draught animals [1]. Additional losses are incurred due to waste of investments made on feed and health measures, and reduced saleable milk production in zebu cows which are known to require a suckling calf for effective stimulation of the milk let-down physiological reflex [2,3]. Interventions aimed at reducing calf mortality have potentially significant benefits on farming enterprises but require specific data on the important causes of mortality and risk factors for each animal production system.

Several studies in East Africa have pointed to multifactorial causes of calf mortality within smallholder systems, mainly related to maternal factors including genetics and mothering abilities, farm management practices, and to infectious agents [1,4-6]. A systematic literature review on causes of morbidity and mortality among smallholder dairy farms in Eastern and South Africa identified tick-borne diseases, diarrhoea and trypanosomiasis as the most commonly documented causes of mortality [7].

Although these studies have generated useful data on risk factors and mortality rates, most have been cross-sectional and not useful in establishing the sequence of events and relationships between infection and mortality. Further, the few longitudinal studies conducted have largely focused on single-pathogen infection systems even in populations known to be commonly co-infected by multiple infections. It is increasingly evident that co-infections, including the numbers and virulence of infecting pathogens, order of infection, infection doses, and interactions between co-infecting pathogens influence the epidemiology of these pathogens, host susceptibility and impacts on infected hosts [8-10].

Among indigenous zebu cattle production systems, epidemiological data that can be used to rank risk factors and different infections in order of importance are largely lacking. Better knowledge of impacts of pathogens on survival probabilities of such hosts could potentially improve the design of disease control strategies, and ultimately their effectiveness.

This study identifies and ranks the risk factors and the main aetiological causes of infectious disease mortality in zebu cattle within the first year of life, raised under smallholder mixed farming system. Specifically, the study a) estimates mortality rates and periods of increased risk for mortality in zebu cattle up to one year of age, b) identifies factors at birth that predict survival of calves and which would be a target for programs aimed at reducing calf mortality, c) identifies infectious and non-infectious risk factors associated with calf mortality, and d) determines the definitive aetiological causes of mortality through a review of post-mortem examination data and results.

## Methods

### Study population

**Figure 1:**
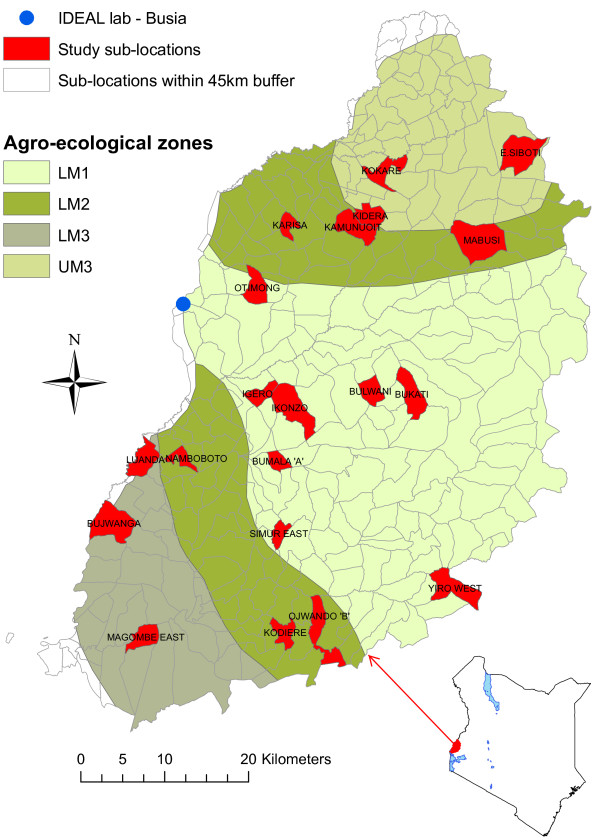
**Map of Western Kenya showing the 4 agro-ecological zones and the 20 study sub-locations (in red).** The study area shown comprised sub-locations falling within a 45 km radius from Busia town on the Kenyan side, where the IDEAL project lab was located. The 4 agro-ecological zones are low midland 1 (LM1), low midland 2 (LM2), lower midland 3 (LM3) and upper midland 3 (UM3).

This study, conducted between October 2007 and September 2010, followed 548 East African shorthorn indigenous zebu calves from birth until one year old. The study area covered sub-locations (smallest administrative units in Kenya) in Western Kenya falling within a 45 km radius of Busia town, where the study field laboratory was located, and comprising 4 agro-ecological zones. A stratified 2-stage random cluster sample of calves was drawn. The 1st stage cluster sample (by sub-location) was selected by random sampling with replacement within each agro-ecological zones stratum and a total of 20 sub-locations, Figure 1. The agro-ecological zones are based on temperature belts and growth suitability of leading crops [11]. A sample size of 28 calves per sub-location was chosen to achieve the desired sample size of 500 calves and to allow for some losses. The recruitment period ran between October 2007 and September 2010. During each recruitment cycle in a sub-location, 1 to 3 calves were randomly selected from all newborns at the time and recruited into the study.

Most farmers in the region practice smallholder mixed crop-livestock production system. An average farm is 2 hectares in size, grows food crops and keeps 5 cattle. The inclusion criteria required that the calves were recruited into the study within a week of birth, be born to a dam that had been on the farm for at least one year and not conceived through artificial insemination. Additionally, only one calf per farm would be in the study at any one time, and the herd should have been under extensive rearing and non-stall feeding. Following recruitment into the study, routine monitoring of study calves was done at 5 week intervals until a year old or until death.

### Data collection

At the recruitment visit and during each of the 5 week routine visits, a complete clinical examination was conducted on each study calf. Clinical samples including blood smears, whole blood and serum samples, faecal samples were collected for screening of pathogens, and measurement of clinical parameters such as total serum proteins and packed cell volume. Blood smears were examined using microscopy. Serum samples collected at every calf visit were screened for antibodies against four main tick-borne diseases; *Anaplasma marginale*, *Babesia bigemina*, *Theileria mutans* and *Theileria parva*. Screening for viruses was carried out on samples collected at one year and included ELISA tests for antibodies against Blue Tongue Virus (BTV), Epizootic Haemorrhagic Disease Virus (EHDV), Infectious Bovine Rhinotracheitis (IBR), Bovine Viral Diarrhoea Virus (BVDV) and Bovine Para- influenza Virus type 3 (PIV3) [12]. Diagnosis of *Ehrlichia ruminantium* was carried out at post-mortem, by examining a brain smear. For helminth diagnosis, McMaster counting technique was used for the identification and quantitative scoring of coccidian oocytes, nematode and cestode eggs per gram of faeces. Additionally faecal cultures were used to differentiate among nematode species [13]. Live body weight measures (in kgs) and girth measurements (in cms) of study calves were also recorded. During each visit, pre-tested questionnaires capturing data on farm characteristics, management practices, herd health, veterinary interventions in the herd, and cattle entries and exits were administered by 5 well trained animal health assistants. Data on the dam of each study calf including its general body health, udder health, girth measurements and body condition score were also recorded, at each calf visit. These data on the dam were collected until the study calf was weaned or until leaving the study at one year.

### Outcome variable

In this paper, the outcome measure of interest was *infectious disease mortality* (ID-mortality): defined as any death of a study calf occurring during the one year observation time and attributed to an infectious disease cause.

### Post-mortem analysis

To determine the specific aetiological cause of death for each case, a standardised post-mortem (PM) examination was conducted, following a standard body system by body system veterinary autopsy routine [14] on calves that died or that were euthanized during the follow-up time. Blood, lymph node and brain smear samples were collected for parasitological examination for bacterial, rickettsial and protozoan parasites. Bacteriological and helminth analysis were carried out on faecal samples collected at PM as described in the data collection section above. Tissue samples from the intestines, lung, liver, spleen, kidney, and heart were collected and fixed in 10% formalin for histopathology examination. Where necessary and dependent on suspected aetiologic causes, additional samples were collected and submitted to the Department of Veterinary Tropical Diseases, University of Pretoria, alongside the samples for histopathological analysis. Results from the laboratory tests, gross and histopathology examinations for each post-mortem case were reviewed by a team of 7 veterinarians and a diagnosis of the main aetiological cause of death for each case determined.

### Risk factors for mortality

These were divided into two main groups: a) non-infectious factors comprising of variables related to the farmer demographics, farm management practices, maternal factors, environmental effects and calf factors, and b) infectious factors being the protozoan, helminth and fungal infections identified from samples collected during the recruitment and 5 week routine visits. Bacterial and viral infections were not included in this analysis because their screening was only done in samples collected during clinical episodes and in the last visit at one year. Table 1 presents a list of the infectious and non-infectious risk factors for calf-mortality tested in the current paper.

**Table 1:** List of covariates tested for their relationship with the infectious disease mortality

	
Farm factors	Farmer’s age, gender, education level, main occupation, herd size, land size
Management factors	Tick control, worm control, trypanosome control, vaccine use, grazing practices, watering practices, housing
Maternal status	Heart girth measurement, body condition score, suckling, health condition, dam antibody titres against *Theileria parva, Theileria mutans, Anaplasma marginale, Babesia bigemina*
Environmental variables	Normalised difference vegetation index (NDVI), farm altitude (elevation)
Calf factors	Calf sex, birth weight, heterozygosity, European introgression, clinical episodes, total serum protein, packed cell volume, white blood cell counts
Infectious factors	Protozoan: *Theileria parva, Theileria mutans, Anaplasma marginale, Babesia bigemina, Trypanosoma* spp., *Coccidia* spp. Helminths: *Calicophoron* spp., *Cooperia* spp., *Dictyocaulus viviparous*, *Fasciola* spp., *Haemonchus placei., Moniezia* spp., *Microfilaria* spp., *Nematodirus* spp., *Oesophagostomum radiatum, Toxocara vitulorum, Trichostrongylus axei, Trichuris* spp., Strongyloides eggs, Strongyle eggs. Fungi: *Trichophyton* spp.

### Statistical analysis

Survival time for each calf was defined as the age at which the calf died due to an infectious cause(s). Animals that died for reasons other than an infectious cause, or that were lost or removed from the study before one year for non-compliance were censored after the last visit with valid data. These animals effectively contributed “at-risk” time only up to the censoring point. All survivors to one year were censored at the time of leaving the study.

Kaplan-Meier estimates of the survival function were used to determine the overall mortality rates [15]. These estimates measured the probability of a calf surviving up to a time *t*, provided by the product of the probabilities of survival at each risk interval prior to time *t*. The survival probability *S(t)* at any particular time *t* is given by Equation 1.

(1) $$S\left(t\right)=\frac{r\left(t_{j}\right)-d\left(t_{j}\right)}{r\left(t_{j}\right)}.$$

Where *t*_*j*_ represents a time interval, *r(t*_*j*_*)* is the number at risk at the start of interval time *t*_*j*_, *d*(*t*_*j*_) is the number of events (deaths) occurring during *t*_*j*_.

To investigate the pattern of risk over the first year of life, instantaneous hazards of failure based on the Kaplan-Meier survival function were used. This measure gives the proportion of the population dying per unit time (Equation 2) and describes the instantaneous probability of an event occurring at a point in time given that it did not occur previously [16]

(2) $$h\left(t\right)=\lim_{\mathrm{\Delta t}\to 0}\frac{P\left(t\leq T<t+\mathrm{\Delta t}|\geq t\right)}{\mathrm{\Delta t}}$$

*h(t)* is the hazard function defined as the probability *P* that a calf dies in a small interval of time *∆t*, given that it survived up to the beginning of the small interval *∆t*, when the size of the time interval approaches zero $\lim_{\Delta \to 0}$. *T* is the calf’s survival time. An R function *epi.instanthaz* in the *epiR* package of **R**[17] was used to calculate the instantaneous hazard rate. Kernel-smoother lines, that estimate average values by aggregating neighbouring point estimates, were added to the instantaneous hazard plots to aid in visualisation of changing risk estimates and reveal the underlying shape of the hazard function.

To overcome limitations associated with standard regression analysis for estimating effects of factors associated with mortality, Cox regression models were favoured. These models utilise information from both censored and non-censored observations [18], and have been extended to incorporate frailty terms and time-varying predictors [19], making it possible to study effects of, for example, infections which are absent at the start of a study and occurring at some point during the study observation time. These Cox proportional hazard models were used to determine the effect of infectious and non-infectious factors on calf mortality. A frailty term for sub-location was included in the models to adjust for clustering within sub-locations. The model used for the analysis is described in Equation 3.

(3) $$h_{i}\left(t\right)=h_{0}\left(t\right)e^{\mathrm{\beta X}+\epsilon_{i}}$$

It expresses the *hazard* at time *t* (i.e. the probability of calf death at time *t*) as a function of:

a) *Baseline hazard* - *h*_0_(*t*): the value of the hazard when all predictors are 0 or absent. It is an unspecified baseline hazard rate describing the common shape of survival time distribution for all calves.

b) *Linear combination of predictors* - *βX*: an exponential function of a series of explanatory variables *βX = β*_*1*_*X*_*1*_ *+ β*_*2*_*X*_*2*_ *+ … + β*_*k*_*X*_*k*_. Their parameters represent the shift in the *log hazard* associated with a unit difference in the corresponding predictor.

c) *Random effect term* – *ϵ*_*i*_: a random effect accounting for the correlated measurements of animals within the same sublocation (shared frailty for the *i*^*th*^ sublocation).

The hazard ratio (HR) was used to determine the effect of covariates, by comparing HRs of groups of calves with different covariate combinations. A covariate with a HR < 1 was interpreted as having a protective effect against mortality, and a covariate with HR > 1, as increasing the risk of mortality. The hazard ratio is the exponential of the Cox regression estimates. For continuous variables, the *hazard ratio* represents a shift in the hazard that is due to a unit change in the predictor whereas for binary predictors, it represents the effect of the factor being present compared to its absence in calves.

To accommodate time-varying predictors in the Cox models, the data were structured such that each calf’s observation time consisted of a series of (*start, stop*) “*intervals of risk*” corresponding to the routine monitoring visits. A calf recruited at 4 days old and with routine visits at age 39, 74 and 109 days will have intervals of risk of (*4,39*], (*39,74*] and (*74,109*]. For each interval, the event of interest (death) is recorded as occurring or not within this period of risk. The survival probability within the intervals of risk is associated with values of covariates as measured at the visit corresponding to the *start* time of each interval. The closed bracket on the right is used to indicate that in case of an overlap, eg. event occurring at day 74, the risk computations will involve the former interval and not the later [19].

Each potential risk factor in Table 1 was initially tested in the model as a univariable analysis. Factors with a *p*-value ≤ 0.2 were then included together in a multivariable analysis. Backward selection procedure was then carried out until only covariates significant at *p*-value < 0.05 remained in the model, referred to as the minimum adequate model (MAM). Predictors dropped during backward selection were added back to MAM one at a time to determine if any significantly improved the model fit. Checks of model fit were done by graphical procedures through examination of residual plots. The analysis in this study was carried out using statistical software R [17] and R package survival [20].

### Ethics statement

The study was reviewed and approved by the University of Edinburgh Ethics Committee (reference number OS 03-06), and also by the Animal Care and Use Committee (AUCUC) of the International Livestock Research Institute, Nairobi. Standard techniques were used to collect blood and faecal samples for diagnosis and identification of disease and infecting pathogens. The calves were restrained by trained animal health assistants, and by veterinary surgeons. A veterinary surgeon was available to examine any calf falling sick during the course of the study. Calves in severe distress due to trauma or disease were humanely euthanised by intravenous injection of sodium pentobarbital by a veterinary surgeon. All participating farmers gave informed consent in their native language before recruiting their animals into the study.

## Results

### All-cause and infectious-disease (ID) mortality

**Figure 2:**
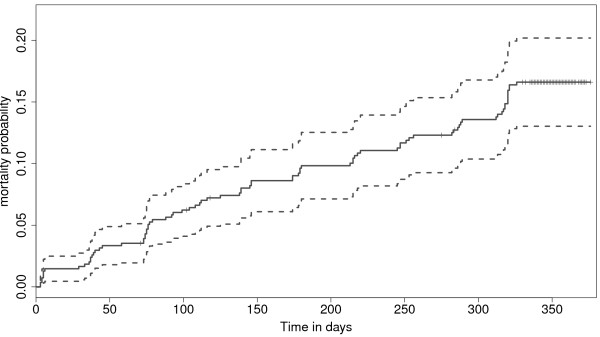
**Kaplan-Meier cumulative risk curve for calf mortality during the first year of life.** The cumulative probability of mortality at one year was estimated at 0.161 CI [0.130 - 0.192].

The 548 animals recruited and followed in the study contributed a total of 175,732 calf days (equivalent to 25,104 calf weeks or 481.1 calf years) of observation. A total of 88 calf deaths were observed. The all-cause mortality rate was estimated at 16.1 (13.0-19.2; 95% CI) per 100 calf years at risk, represented by the Kaplan-Meier curves in Figure 2. Based on the history before death, 5 of the 88 deaths were considered to be non-infectious and censored in the analysis of infectious-disease related deaths (ID-mortality). The non-infectious causes of death included trauma, plant poisoning and starvation. For logistical reasons, PM’s were not carried out on 6 of calves that died and their cause of death could not be determined. The remaining calves were treated as having died from infectious diseases. The estimated minimum ID-mortality rate was 13.3 (10.4-16.3; 95% CI) per 100 calf years at risk.

**Figure 3:**
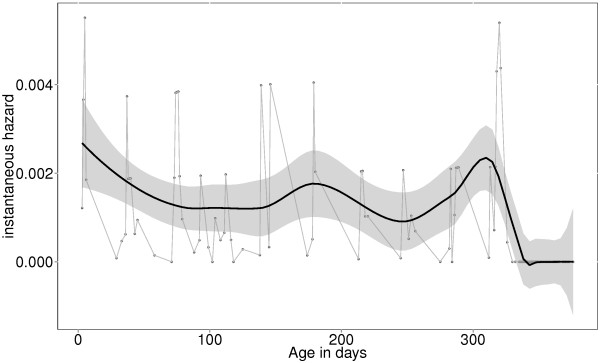
**Instantaneous hazard estimates with kernel-smoothing for calves under one year.** The plot shows three periods of increased risk for mortality: period immediately after birth (neonatal period), period between 150 and 190 days (corresponding to expected time for waning maternal immunity), and period towards one year of age (corresponding to age of weaning).

The temporal risk pattern for ID-mortality was determined using estimates of the instantaneous risk calculated using Equation 2. The results were plotted and Kernel-smoothing lines added to aid visualization and identification of periods of increased risk for ID-mortality, see Figure 3.

### Spatial pattern of mortality

**Figure 4:**
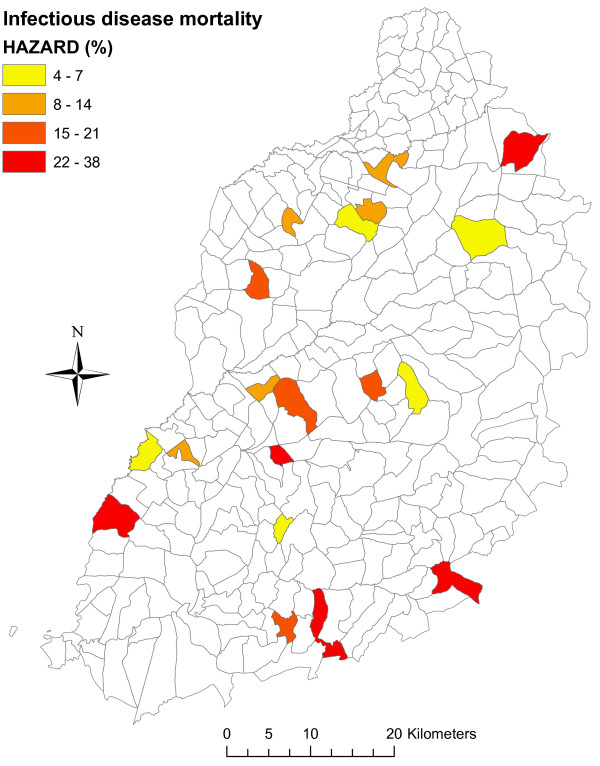
**Choropleth map showing mortality rates by study sub-location.** Higher mortality rates observed in sub-locations in the South, and lower rates in sub-locations towards North. The variable “Northing” is marginally statistically associated with calf mortality (*p*-value = 0.078). The north most sub-location (East Siboti) has a high mortality and masks the observed association between mortality and northing (*p*-value = 0.007, when East Siboti is omitted).

To explore the spatial patterns in mortality, mortality rates for each study sub-location were determined and plotted as a choropleth maps, Figure 4. The mortality rates between sub-locations ranged from as low as 3.6 to as high as 38.5 per 100 calf years, see Table 2. The log-rank test for differences in Kaplan-Meier curves of the study sub-locations was statistically significant (*p*-value = 0.01). Mortality rates were higher in the sub-locations falling within the southern region of the study area compared to those in the northern region, except for the north most sub-location (East Siboti) which recorded the second highest mortality. Kaplan-Meier curves for the 20 study sub-locations revealed temporal differences in mortality between sub-locations. Results showed for example that in Bumala A and Magombe East (sub-locations lying in the South) died at a relatively young age (< 150 days, < 220 days respectively), compared to those in East Siboti (in the North) where death occurred at a relatively older age. This pattern may be related to the aetiological cause of death, with most deaths in Bumala A and Magombe East attributed to East Coast Fever, whereas those in East Siboti were mainly due to haemonchosis. The median survival time for calves that died due to ECF was 93 days CI [73-146], while that for calves that died due to haemonchosis was 271 days CI [125 - NA (> 365 days)].

**Table 2:** Results of Kaplan-Meier analysis showing the survival probabilities and their 95% confidence intervals for calves by study sub-location

**Sub-location**	**No. at start**	**No. of deaths**	**Survival**	**std.err**	**Lower CI**	**Upper CI**
Bujwanga	28	6	0.783	0.0786	0.643	0.953
Bukati	28	1	0.964	0.0351	0.898	1
Bulwani	28	5	0.821	0.0724	0.691	0.976
Bumala A	22	6	0.727	0.0950	0.563	0.939
East Siboti	28	8	0.714	0.0854	0.565	0.903
Igero	28	3	0.893	0.0585	0.785	1
Ikonzo	28	4	0.857	0.0661	0.737	0.997
Kamunuoit	27	2	0.926	0.0504	0.832	1
Karisa	28	3	0.891	0.0592	0.783	1
Kidera	28	3	0.891	0.0592	0.783	1
Kodiere	27	4	0.852	0.0684	0.728	0.997
Kokare	28	4	0.857	0.0661	0.737	0.997
Luanda	28	2	0.929	0.0487	0.838	1
Mabusi	28	2	0.929	0.0487	0.838	1
Magombe East	26	10	0.615	0.0954	0.454	0.834
Namboboto	27	3	0.889	0.0605	0.778	1
Ojwando B	28	7	0.750	0.0818	0.606	0.929
Otimong	28	6	0.786	0.0775	0.648	0.953
Simur East	27	1	0.963	0.0363	0.894	1
Yiro West	28	6	0.780	0.0794	0.639	0.952

### Risk factors for mortality

To investigate the risk factors associated with calf mortality, the analysis was carried out to determine the following a) risk factors for ID-mortality using data collected at recruitment time (predictors at birth), b) non-infectious and infectious risk factors for calf mortality over time.

### Predictors at birth

Putative factors collected at recruitment time were initially tested in a univariable analysis (results shown in Additional file 1: Table S1). While including sub-location as a random effect, factors from univariable analysis with *p-*values ≤ 0.2 were offered to the multivariable models. Three variables; a) watering at homestead which represents farms in which drinking water was provided at the homestead rather than driving animals a distance away from the homestead for watering, b) antibody titres against *T.parva* and c) *B.bigemina* in the dams were statistically associated with ID-mortality, see results in Table 3. Watering at homestead was associated with a 50% (31, 80; 95% CI) decrease in the hazard for ID-mortality. High antibody titres against *T.parva* and *B.bigemina* in the dams at recruitment were associated with increased ID-mortality hazard by a factor of 1.13 (1.04, 1.23; 95% CI) and 1.11 (1.03, 1.20; 95% CI) times for every 10 unit increase above the mean titres for *T.parva* and *B.bigemina* respectively. The standard deviation of the random effect is interpreted as: one standard deviation above the mean corresponds to a relative risk exponential (0.39) = 1.48, a 48% higher risk of death for calves in that sub-location [21].

**Table 3:** Predictors of mortality at calf recruitment time

	**Hazard ratio**	**Lower CI**	**Upper CI**	***p-*value**
Fixed effects				
Watering at homestead	0.50	0.31	0.8	0.003
*(T.parva* antibodies/10)-dam	1.13	1.04	1.23	0.004
*(B.bigemina* antibodies/10)-dam	1.11	1.03	1.2	0.007
Random effects				
Group	Variable	Std Dev	Variance	
Sub-location	Intercept	0.39	0.15	

### Predictors over time of ID-mortality

Non-infectious and infectious factors recorded in Table 1 were initially evaluated as univariable analysis. Factors with a *p-*value ≤ 0.2 which were selected to be included in the multivariable analysis (see Additional file 2: Table S2). Model simplification was done by through backward selection, until only variables significant at *p*-value < 0.05 remained in the model.

The model identified *Theileria* spp. infections, the most lethal of which causes East Coast Fever disease, infection with *Trypanosoma* spp. (the main species being *Trypanosoma vivax*), and high strongyle eggs per gram count as the three important infections with a statistically significant association with ID-mortality in calves. While holding other covariates constant, high intensity *Theileria* spp. infections (i.e. level 3 infections - defined as ≥ 2 infected cells per microscopy field, in multiple fields), infection with *Trypanosoma* spp., and an increase in strongyle epg count by 1000 increased the hazard for ID-mortality by a factor of 39, 8 and 1.5 times respectively. *T.parva* seropositivity was associated with a protective effect, with calves that had seroconverted having a reduced risk of ID-mortality by 88% compared to animals that did not seroconvert. Controlling for ticks in the farm and providing drinking water to the animals at the farm were both associated with a protective effect against ID-mortality. Tick control was associated with a 49% lower mortality hazard compared to farms that did not control for ticks. Watering at homestead reduced the risk of ID-mortality by 65% compared to farms where watering was not done at the homestead. Results shown in Table 4.

**Table 4:** Results of the minimum adequate survival model with significant variables associated with calf mortality

	**Hazard ratio**	**Lower CI**	**Upper CI**	***p-*value**
Fixed effects				
Tick Control	0.51	0.27	0.96	0.036
Watering at homestead	0.35	0.18	0.67	0.001
*T.parva* seropositivity	0.12	0.06	0.23	< 0.001
*Theileria* spp. Level 1	0.67	0.34	1.32	0.25
*Theileria* spp. Level 2	2.43	0.74	8.03	0.14
*Theileria* spp. Level 3	38.85	6.62	228.02	< 0.001
*Trypanosoma* spp.	7.90	1.80	34.95	0.006
(Strongyle epg/1000)	1.52	1.39	1.68	< 0.001
Random effects				
Group	Variable	Std Dev	Variance	
Sub-location	Intercept	0.0199	0.0004	

**Figure 5:**
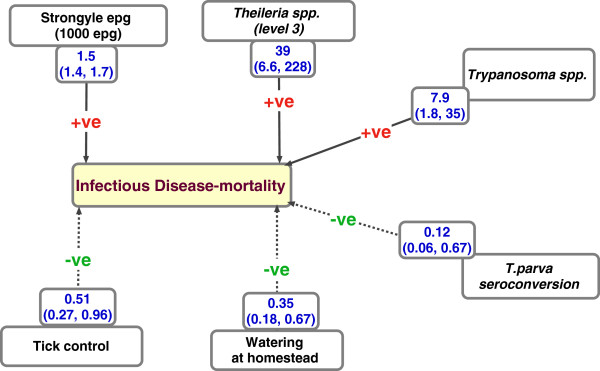
**Schematic diagram showing the summary results of predictors for infectious disease mortality (ID-mortality), with the size of effect.** The estimated ID-mortality was 13.3 (10.4-16.3; 95% CI) per 100 calf years at risk. The variables with a -ve sign on the lines have a protective effect against ID-mortality. The main pathogens identified to be associated with increased risk for death are infection with *Trypanosoma* spp. mainly being *T.vivax*, high intensity infection with *Theileria* spp. as observed at microscopy, and high worm burden measured by the strongyle eggs per gram of faeces. Seroconversion to *T.parva* was associated with a protective effect.

The diagnostic plots of scaled Schoenfeld residuals [19] showed that the assumption of proportional hazard was supported by all the variables within the model, except for the variable “watering at homestead” whose effect decreased with age of calf. The non-proportional hazard for this factor (watering at homestead) was accommodated in the model by stratifying the data based on the two levels (Yes and No) for this variable. This assumes each stratum has a different baseline hazard function, while the other covariates are assumed to be constant across strata. The results of this model showed no evidence of non-proportionality in any of the remaining covariates, and which remained significantly associated with ID-mortality. A summary schematic diagram showing the predictors, their effect sizes and direction on the outcome ID-mortality is provided in Figure 5.

### Cause-specific mortality

**Figure 6:**
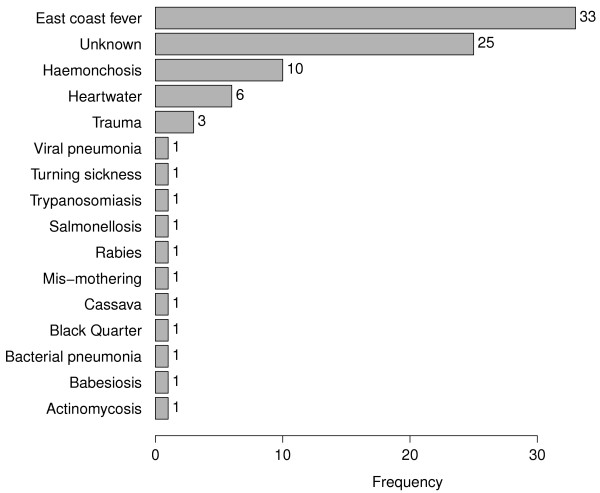
**Definitive aetiological causes of death.** A total of 88 deaths occurred during the study. Five of these were attributed to non-infectious causes (trauma, mis-mothering and cassava poisoning). East Coast Fever was the main cause of death, followed by haemonchosis and heartwater disease. For 25 deaths, a definitive aetiological cause of death could not be determined.

The definitive aetiological causes of mortality based on the post-mortem analysis are presented in Figure 6. The main cause of death was identified as East Coast Fever (ECF), accounting for 40% of all deaths due to infectious diseases (6% crude mortality). The second major cause of mortality was haemonchosis, attributed to heavy infection with *Haemonchus placei*, a hookworm that attaches to the abomasal wall sucking whole blood. Haemonchosis was identified as the cause of 12% of the ID-mortality (1.8% crude mortality). Heartwater disease was identified as the third major cause of ID-mortality, accounting for 7.2% of the infectious disease deaths (1.1% crude mortality).

The aetiological causes of death varied between sub-locations with deaths due to haemonchosis being observed more in the North (East Siboti sub-location) whereas most ECF deaths were observed in the South (Magombe East and Bumala A sub-locations).

Although a definitive aetiological cause could not be determined for 29% of the mortality cases, contributing infectious causes for many of these cases were identified. Over half the ECF deaths were complicated by other infections, helminthiasis being the main co-infection. Although trypanosomiasis and anaplasmosis were not identified as definitive causes of calf mortality for any case, they were important co-infecting pathogens for some of the deaths attributed to haemonchosis and ECF.

## Discussion

This study has investigated mortality in indigenous zebu cattle during their first year of life, and identified the main aetiological causes of death and the risk factors associated with infectious disease mortality. The all-cause mortality rate was estimated at 16.1 per 100 animal risk years, while mortality related to infectious diseases (ID-mortality) was estimated at 13.3 per 100 animal risk years. Although zebu cattle are considered well adapted to survive in environments of high disease pressure [22], mortality rates as observed in this study would suggest significant losses. These mortality rates are more than 3 times what is observed in most well managed dairy systems in developed countries where the all-cause mortality rates are frequently reported to be below 5% [23-25]. Unlike dairy systems which have intensive management and veterinary input, the traditional production systems under which zebu cattle are raised are largely non-interventional with little or no disease control at the farm level.

Similar mortality rates to those observed in this study have been reported among zebu calves in Tanzania [6,26]. The review by Otte and Chilonda focussing on the production parameters among cattle raised under different agro-ecological zones and production systems in Sub-Saharan Africa reported an overall calf mortality risk of 21.7 percent in traditional smallholder mixed production systems [27]. Among exotic and cross-bred animals in Sub-Sahara Africa, the overall mortality rates are usually higher than those observed in zebus, with some studies reporting rates as high as 35% [1,4,28]. From these studies it is evident that although zebu cattle have relatively lower mortality compared to exotics and cross-bred animals, calf mortality is still high and a possible significant impediment to improved livestock production.

In order to establish if there were specific periods when calves were at relatively higher risk of death, this study used the instantaneous hazard estimates plotted with kernel-smoothing to aid visualisation (Figure 2). The diagram suggests three periods of relatively higher risk for calf ID-mortality: neonatal period, age between 5 and 6 months (corresponding to period of waning maternal antibodies), and age approaching one year (corresponding to weaning time). Most studies on calf mortality identify the neonatal period (first four weeks after birth) as the period with highest risk for calf mortality [23,24,29,30]. Diarrhoea and pneumonia are frequently reported to be the main causes of death during this period, with inadequate or delayed ingestion of colostrum after birth, unhygienic manual feeding of milk to the calves, poor calf feeding and poor housing being the main risk factors associated with these deaths. In this study, diarrhoea and pneumonia were uncommon and were not identified as causes of death for any study animals. Zebu calves are allowed to suckle directly from the dams, which reduces the risk of hygiene related illnesses associated with manual feeding of milk to the calves.

Initially the study sought to establish the predictors of ID-mortality at birth. High antibody titres against *T.parva* and *B.bigemina* in the dam were associated with increased risk for death. One possibility may be that the antibody titres may be a form of measure of infection pressure in the location the calf is born, with higher titres indicating higher infection pressure. Their effect was lost in the subsequent models that included calf infection data.

The husbandry practice of providing drinking water to the animals within the homestead was found to have a protective effect against ID-mortality in the recruitment model and the model with infectious and non-infectious factors observed over the one year. It would be expected that animals that do not require traveling to common watering points for groups of animals from different farms have lower exposure levels to pathogens. This may be one reason why this factor was identified associated with a protective effect against ID-mortality. It would be expected that including pathogen data would reduce the importance of this variable, and it is curious its effect remains with pathogen data included in the model. It is unclear what other variation other than that explained by pathogen data, the variable captures.

Before incorporating infection data into the models, analysis of the non-infectious factors associated with ID-mortality revealed heart girth size, the mean Normalised Difference Vegetation Index (NDVI), watering at homestead, controlling for ticks and dam antibody titres against *B.bigemina* as the significant non-infectious predictors of ID-mortality. The effects of mean NDVI, heart girth size (both protective effects) and antibody titre against *B.bigemina* (increased risk of death) were however lost when infection data was included, indicating these factors may be related to either susceptibility to infections or infection pressure. NDVI measures the health and density of vegetation with high NDVI values indicating healthy vegetation a proxy measure of environmental variables as rainfall and temperature. Since it measures the vegetation health, it may be related with the quality and quantity of feed availability for the animals or with high tick abundance and incidence of tick-borne diseases. High NDVI values may be suggestive of good feed availability to the dam and consequently to a suckling calf, and which would relate to the observed protective effect against calf ID-mortality. The heart girth size in the dam may relate to the condition of the dam and possibly the quality of care extended to the calf mainly through feeding. Absence of tick control and watering at homestead remained significant predictors of calf ID-mortality in the subsequent analysis including infection factors.

Although the study calves were not themselves sprayed with acaricides to control for ticks, tick control in the rest of the herd was associated with a 49% lower risk of death when compared to animals in farms where tick control was absent. Tick control in the herd may reduce the level of exposure to infected ticks that calves experience, thereby improving their survival probabilities. The frequency with which tick control was done within farms controlled for ticks was itself very low, with only a fraction of farms doing tick control more than two times in the year. This raises a question whether occasional tick control, even though it may not keep the cattle completely tick free, still carries some benefits especially in relation to survival of calves in the herd.

The final Cox survival model identified high intensity infection with *Theileria* spp. observed at microscopy, infection with *Trypanosoma* spp. and high strongyle faecal egg counts to significantly increase the risk of ID-mortality by a factor of 39, 8, and 1.5 (per 1000 epg increase) respectively. The model used data obtained through microscopy and did not include information such as clinical history (signs before death), gross or histo-pathology findings following post-mortem analysis.

When compared to the results obtained from the independent systematic review of laboratory, gross-pathology and histo-pathology data of all post-mortem cases done by the 7 veterinarians to establish the definitive aetiological causes of death for each case, the findings have good agreement. The review of the post-mortem examination results revealed, in order of importance, the main causes of calf mortality to be East Coast Fever, haemonchosis and heartwater disease. These three infections directly accounted for 60% of the disease-induced mortality. Larval cultures routinely carried out to identify the species of worms infecting the calves revealed that *Haemonchus placei* accounted for > 80% of all larvae hatched from strongyle eggs. The presence and abundance of *H.placei* worms was confirmed at post-mortem.

Although heartwater disease was identified as a main cause of death through the review of post-mortem examination results, it would not be possible to predict this since *E.ruminantium*, the causative agent for heartwater disease, is not easily detected in blood. Diagnosis of heartwater is mainly through clinical signs, although deaths may be per-acute, and confirmation by demonstration of *E.ruminatum* bodies in brain smears prepared during post-mortem examination. The model identifies infection with *Trypanosoma* spp. as significantly associated with death. From the PMs, trypanosomiasis was identified as the main cause of death for one calf and as a contributing cause of death to other cases.

The diagnosis of the three main infections (*Theileria* spp., *Trypanosoma* spp., and strongyle epg) identified important by the model is done on microscopy which is easily applicable in the field. It is also not labour intensive and requires little time to complete pointing to opportunities of applying simple diagnostic techniques whose results would help significantly reduce calf mortality.

The causes of calf mortality vary between geographical regions and production systems. Within smallholder production systems, some studies report pneumonias, digestive tract disorders including non-parasitic diarrhoeas, bloat [1,5,29,30], and tick-borne diseases (TBD) [4,6,31] as the major causes of mortality. Although the current study covered a region within a 45 km radius semicircle from Kenya-Uganda border, differences in mortality rates and patterns between study sub-locations were evident. Higher mortality estimates were observed in sub-locations in the southern region of the study area and occurred in relatively younger animals compared to those in the northern region. These differences corresponded to the aetiological causes of death, with ECF being the main cause of death in the South and haemonchosis in the North. Such spatial heterogeneity within relatively small regions demonstrates the need for evidence based design for the control of disease and reduction of calf mortality.

The importance of the transfer of maternal antibodies into neonate calves via colostrum is known to be associated with survival chances of neonates [32]. This is especially important in ruminants where very little transfer of such antibody occurs in-utero and where the ability of the newborn calf to absorb colostral antibodies is limited to the first few hours of life. It is important to note colostrum uptake was not directly measured in the study, as the calves were recruited 3 to 7 days after birth. Although the quality and amount of colostrum ingested was not established, data on whether the calf suckled immediately after birth was included in the analysis. In addition, antibody titres against the four main tick-bornes (*T.parva, T.mutans, A.marginale* and *B.bigemina*) in the dam were included in the analysis.

## Conclusions

From this study, providing animals with drinking water at the homestead as opposed to accessing water from communal watering points, and controlling for ticks within the farm have been identified as the two important husbandry practices that may decrease the risk of calf ID-mortality. When compared to farms where animals do not access drinking water at the homestead, and where tick control is not practised, these two husbandry practices would be estimated to decrease the risk of mortality by 65% and 50% respectively. It is interesting to note however that the protective effect identified is from relatively infrequent tick control, and not the intensive methods used in dairy systems. Tick control would reduce the risk of death due to ECF and heartwater diseases, both of which are tick-borne diseases. An additional method for the control of ECF would be the immunization through the Infection Treatment Method (ITM) which is currently available for use in most of the country. Decisions on treatment of ECF cases can be aided by microscopy results as this study has shown that high infection intensities at microscopy are a strong predictor for calf mortality. Similarly, strongyle epg count in this case was identified as a good predictor for calf death, and can be used for epidemiological purposes as well as decision-making at the farm level.

## Competing interests

The authors declared that they have no competing interests of any kind in the study design, collection, analysis and interpretation of data, writing and submission of the manuscript for publication.

## Authors’ contributions

WMEJ, B BMdeC, CK, HO, KH, TP conceived the study, participated in its design and coordination, interpretation of results and helped draft the manuscript. TSM collected, analysed the data, interpreted results and drafted the manuscript. PEJ and HIG participated in the design of the project, database management, offering statistical support and helped to draft of the manuscript. SJCA participated in the histopathological and post-mortem assessments, and helped draft the manuscript. NM, JA and CI participated in the data collection, laboratory and statistical analysis and helped draft the manuscript. All authors read and approved the final manuscript.

## Supplementary Material

Additional file 1: Table S1 Results showing factors offered to the multivariable analysis for factors at birth model for infectious disease mortality. Click here for file

Additional file 2: Table S2 Results showing factors offered to the multivariable model for analysis of risk factors for infectious disease mortality. Click here for file
