# Supplementary material for: Mortality in East African shorthorn zebu cattle under one year: predictors of infectious-disease mortality
Source: BMC Vet Res. 2013 Sep 8;9:175. doi: 10.1186/1746-6148-9-175 (PMC3848692; doi:10.1186/1746-6148-9-175)
Supplement: Additional file 1: Table S1 — Results showing factors offered to the multivariable analysis for factors at birth model for infectious disease mortality. [file 1746-6148-9-175-S1.docx]

Supplementary table

Table S1: Results showing factors offered to the multivariable analysis for factors at birth model for infectious disease mortality

|  | Hazard ratio | se(coef) | z | *p*-value |
| --- | --- | --- | --- | --- |
|  |  |  |  |  |
| Farmer’s age | 1.0165 | 0.0079 | 2.0613 | 0.0393 |
| log(Tropical livestock units) | 1.4619 | 0.1482 | 2.563 | 0.0104 |
| Use supplements – yes | 0.6816 | 0.2510 | -1.5268 | 0.1268 |
| Tick control – yes | 0.6525 | 0.0868 | -4.9215 | < 0.001 |
| Worm control – yes | 0.6843 | 0.2044 | -1.8562 | 0.0634 |
| Milk prior calving | 0.3686 | 0.588 | -1.6976 | 0.0896 |
| Milk post calving | 0.6978 | 0.2381 | -1.5112 | 0.1307 |
| Watering at homested | 0.456 | 0.2288 | -3.4314 | 0.0006 |
| Distance to water - < 1 km | 0.7137 | 0.2348 | -1.4363 | 0.1509 |
| Heterozygosity | 0.0003 | 5.4717 | -1.4661 | 0.1426 |
| Recruitment weight | 0.9546 | 0.0293 | -1.5859 | 0.1128 |
| *B.bigemina* antibodies - calf | 1.0052 | 0.0038 | 1.379 | 0.1679 |
| Total serum proteins | 0.8765 | 0.0838 | -1.5726 | 0.1158 |
| White blood cell count | 0.9528 | 0.0355 | -1.3616 | 0.1733 |
| Heart girth size – dam | 0.9744 | 0.0141 | -1.8384 | 0.066 |
| *T.parva* antibodies – dam | 1.0122 | 0.0042 | 2.8981 | 0.0038 |
| *B.bigemina* antibodies - dam | 1.0123 | 0.0037 | 3.3306 | < 0.001 |
