# Supplementary material for: Mortality in East African shorthorn zebu cattle under one year: predictors of infectious-disease mortality
Source: BMC Vet Res. 2013 Sep 8;9:175. doi: 10.1186/1746-6148-9-175 (PMC3848692; doi:10.1186/1746-6148-9-175)
Supplement: Additional file 2: Table S2 — Results showing factors offered to the multivariable model for analysis of risk factors for infectious disease mortality. [file 1746-6148-9-175-S2.docx]

**SUPPLEMENTARY TABLES**

Table S2: Results showing factors offered to the multivariable model for analysis of risk factors for infectious disease mortality

| Variable | Hazard ratio | se(coef) | z | *p*-value |
| --- | --- | --- | --- | --- |
| Farmer’s age | 1.014 | 0.0083 | 1.6758 | 0.0938 |
| log(Tropical livestock units) | 1.479 | 0.1485 | 2.6348 | 0.0084 |
| Watering at homestead | 0.4542 | 0.2288 | -3.4491 | < 0.001 |
| Distance to water < 1km | 0.7037 | 0.2348 | -1.4964 | 0.1345 |
| Suckling-yes | 0.4286 | 0.3931 | -2.1555 | 0.0311 |
| Milk prior calving | 0.3722 | 0.588 | -1.6809 | 0.0928 |
| Milk post calving | 0.7243 | 0.2385 | -1.3521 | 0.1763 |
| Use supplements – yes | 0.6792 | 0.2510 | -1.5407 | 0.1234 |
| Housing - stall shed | 0.7445 | 0.2159 | -1.3666 | 0.1717 |
| Tick control – yes | 0.3191 | 0.2164 | -5.2795 | < 0.001 |
| Worm control – yes | 0.6561 | 0.2709 | -1.5555 | 0.1198 |
| Antibiotics use – yes | 0.6029 | 0.2348 | -2.1551 | 0.0312 |
| Mean NDVI | 0.006 | 2.3009 | -2.2202 | 0.0264 |
| Heart girth size – dam | 0.9524 | 0.0171 | -2.8537 | 0.0043 |
| Body condition score- dam | 0.7066 | 0.1247 | -2.7865 | 0.0053 |
| Health of dam – sick | 3.7435 | 1.0109 | 1.3058 | 0.1916 |
| Heterozygosity | 0.0003 | 5.4257 | -1.5091 | 0.1313 |
| *T.parva* antibodies – dam | 1.0133 | 0.0042 | 3.1277 | 0.0018 |
| *B.bigemina* antibodies - dam | 1.0122 | 0.0037 | 3.2456 | 0.0012 |
| *Trypanosoma* spp. | 4.2749 | 0.5911 | 2.4576 | 0.014 |
| *Trypanosoma vivax* | 5.5339 | 0.7217 | 2.3706 | 0.0178 |
| *T.parva* – seropositivity | 0.2227 | 0.2819 | -5.3281 | < 0.001 |
| *T.mutans* – seropositivity | 0.4937 | 0.2770 | -2.5486 | 0.0108 |
| *A.marginale* - seropositivity | 0.6573 | 0.3105 | -1.3513 | 0.1766 |
| *Cooperia* spp. | 4.3323 | 0.7336 | 1.9986 | 0.0456 |
| *Moniezia* spp. | 67.0774 | 1.0691 | 3.9342 | < 0.001 |
| *Trichophyton* spp. | 3.7553 | 0.7234 | 1.8292 | 0.0674 |
| *Theileria* spp. Level 2 | 2.6377 | 0.4633 | 2.0935 | 0.0363 |
| *Theileria* spp. Level 3 | 11.243 | 0.7329 | 3.3014 | < 0.001 |
| Strongyle epg/1000 | 1.4164 | 0.0384 | 9.0604 | < 0.001 |
| *Calicophoron spp.* | 1.5363 | 0.2945 | 1.458 | 0.1448 |
